# [Corrigendum] Upregulated NTF4 in colorectal cancer promotes tumor development via regulating autophagy

**DOI:** 10.3892/ijo.2025.5725

**Published:** 2025-02-10

**Authors:** Zhou Yang, Yusheng Chen, Xiyi Wei, Dejun Wu, Zhijun Min, Yingjun Quan

Int J Oncol 56: 1442-1454, 2020; DOI: 10.3892/ijo.2020.5027

Following the publication of the above article, the authors subsequently realized that, during the process of collating the raw data, Fig. 1 [the immunohistochemical (IHC) results for stage IV colorectal cancer (CRC)], Fig. 2A (the control β-actin blots) and Fig. 5C and D (both the images selected for the clone formation assays, and the histograms showing the quantification of the data) were inadvertently assembled incorrectly. These errors arose as a consequence of the affected files having been named similarly to those of the correct panels.

The revised versions of Figs. 1, 2 and 5, now featuring the correct IHC data for stage IV CRC in Fig. 1, the correct control western blots in Fig. 2 and the correct colony formation assay data (and quantification thereof) in Fig. 5, are shown on the next three pages. Note that the correction of these figures does not affect the key findings of the study (either the existing published results or the conclusions reached from the results). The authors thank the Editor of *International Journal of Oncology* for granting them the opportunity to publish this corrigendum. All the authors agree with the publication of this corrigendum; furthermore, they apologize to the readership of the journal for any inconvenience caused.

## Figures and Tables

**Figure 1:**
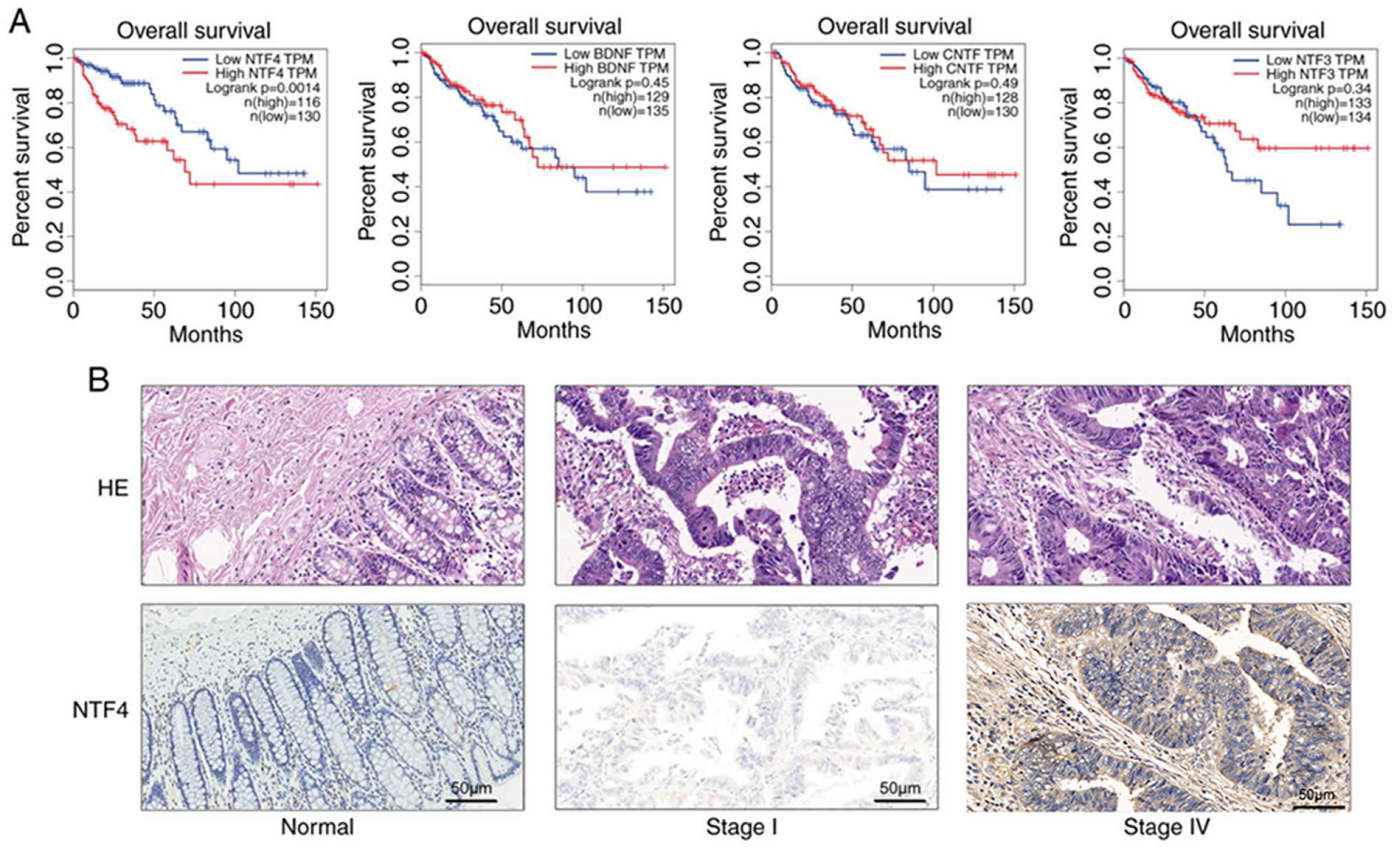
(A) The association between overall survival in patients with CRC and NTF expression was assessed using the GEPIA website tool through Kaplan-Meier analysis. (B) Hematoxylin and eosin (HE) and immunohistochemistry staining of normal tissue and CRC at different stages (magnification, ×200). (C) The protein expression levels of NTF4 were detected using western blotting. (D) The expression levels of NTF4 in normal samples and patients with CRC were analyzed using TCGA database. (E) The expression levels of NTF4 in multiple types of cancer were analyzed using TCGA database. NTF4, neurotrophin-4; CRC, colorectal cancer; TCGA, The Cancer Genome Atlas. ^***^P<0.001.

**Figure 2:**
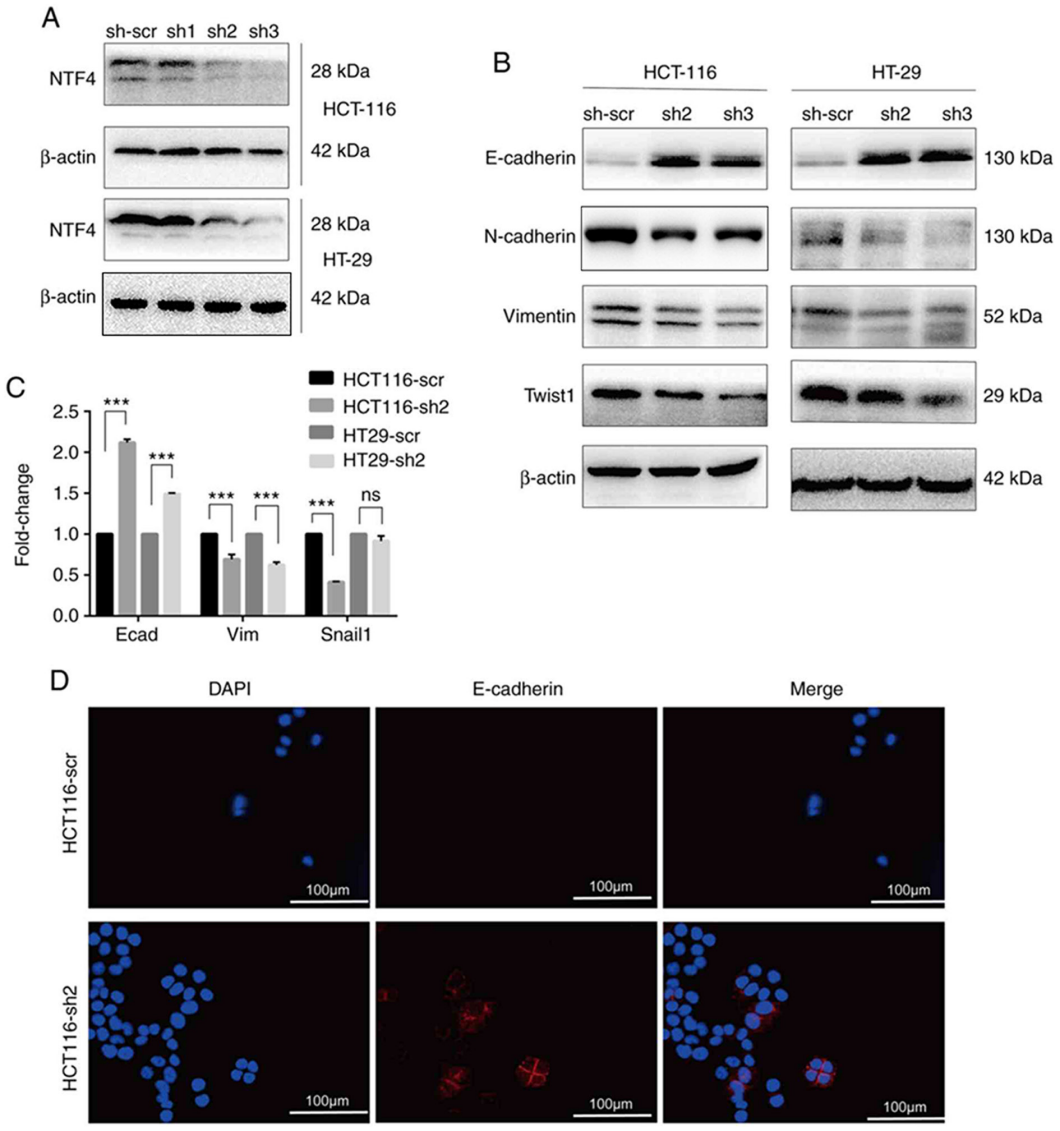
(A) Short hairpin RNA lentivirus transfection in HCT116 and HT-29 cells was confirmed using western blotting. (B) The expression levels of EMT markers in HCT116 and HT-29 cells were detected using western blotting. (C) The mRNA expression levels of EMT markers in HCT116 and HT-29 cells were detected using reverse transcription-quantitative PCR. (D) Localization and expression of E-cadherin in HCT116 cells were detected by immunofluorescence. ^***^P<0.001. EMT, epithelial-to-mesenchymal transition; NTF4, neurotrophin-4; CRC, colorectal cancer.

**Figure 5:**
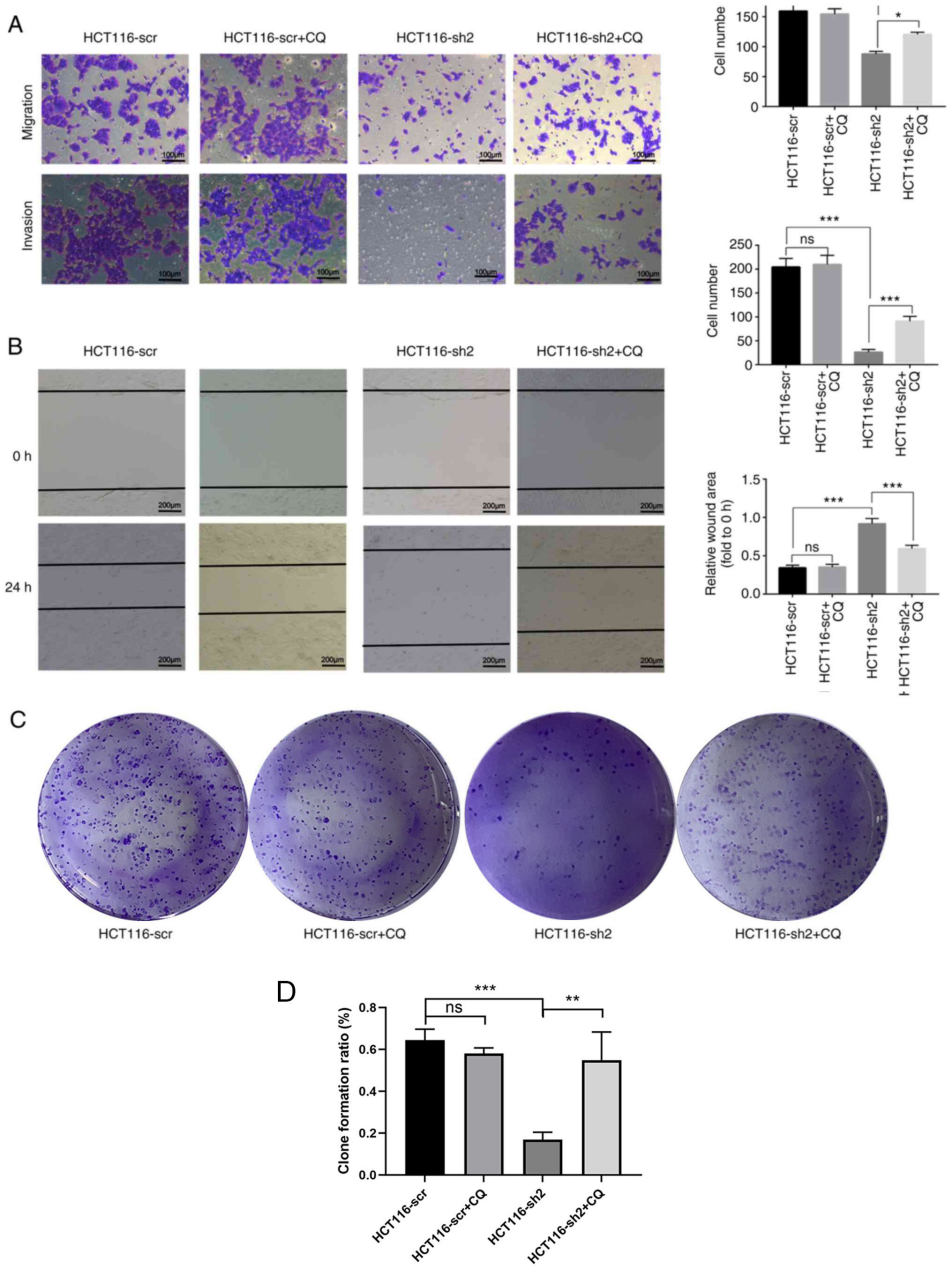
(A) Cell migration and invasion ability was analyzed using Transwell assays. (B) Cell migration ability was analyzed using the wound healing assay. (C) Colony formation assay and (D) ratio of each group. ^*^P<0.05, ^***^P<0.001. (E) Cell proliferation of each group was analyzed using a Cell Counting Kit-8 assay. ^***^P<0.001, HCT116-sh2 vs. HCT116-scr; ^##^P<0.01, ^###^P<0.001, HCT116-sh2 vs. HCT116-sh2 + CQ group. (F and G) Cell cycle analysis of HCT-116 cells using flow cytometry. ^***^P<0.001. sh, short hairpin RNA; scr, scramble; CQ, chloroquine.

